# Avaliação do kit Filaria Detect™ IgG4 produzido com o antígeno recombinante Wb123 para diagnóstico da filariose linfática no Brasil

**DOI:** 10.26633/RPSP.2021.87

**Published:** 2021-07-08

**Authors:** Paula Fernanda A. S. Melo, Maria Almerice L. Silva, Maria Rosângela G. Oliveira, Josué Araújo, Amanda Fernandes, George Tadeu N. Diniz, Eduardo Brandão, Abraham Rocha, Maria Amélia Maciel

**Affiliations:** 1 Universidade Federal de Pernambuco (UFPE), Programa de Pós-Graduação em Medicina Tropical Recife (PE) Brasil Universidade Federal de Pernambuco (UFPE), Programa de Pós-Graduação em Medicina Tropical, Recife (PE), Brasil.; 2 Fundação Oswaldo Cruz (Fiocruz), Instituto Aggeu Magalhães (IAM), Departamento de Parasitologia/Serviço de Referência Nacional em Filarioses Recife (PE) Brasil Fundação Oswaldo Cruz (Fiocruz), Instituto Aggeu Magalhães (IAM), Departamento de Parasitologia/Serviço de Referência Nacional em Filarioses, Recife (PE), Brasil.; 3 Fundação Oswaldo Cruz (Fiocruz), Instituto Aggeu Magalhães, Núcleo de Saúde Coletiva (IAM/NESC) Recife (PE) Brasil Fundação Oswaldo Cruz (Fiocruz), Instituto Aggeu Magalhães, Núcleo de Saúde Coletiva (IAM/NESC), Recife (PE), Brasil.

**Keywords:** Filariose linfática, diagnóstico, anticorpos, *Wuchereria bancrofti*, Brasil, Elephantiasis, filarial, diagnosis, antibodies, *Wuchereria bancrofti*, Brazil, Filariasis linfática, diagnóstico, anticuerpos, *Wuchereria bancrofti*, Brasil

## Abstract

O Plano Global de Eliminação da Filariose Linfática, lançado pela Organização Mundial da Saúde em 2000, propõe o uso de testes de detecção de antígeno circulante filarial como ferramenta diagnóstica para avaliação e monitoramento das ações de controle da parasitose. Entretanto, esses testes, apesar de apresentarem alta sensibilidade, não conseguem detectar com eficiência a infecção em seu estágio inicial, quando ainda não existe a presença de helmintos adultos. Considerando essa limitação, a pesquisa de anticorpos antifilariais tem sido apontada como uma alternativa, uma vez que os anticorpos produzidos contra as larvas infectantes do parasito são detectados antes da presença de antígeno circulante filarial. O objetivo deste estudo foi definir o ponto de corte e avaliar a acurácia do kit Filaria Detect™ IgG4 produzido com o antígeno recombinante Wb123 para diagnóstico da filariose linfática no Brasil. Para isso, foi realizado um estudo de avaliação de teste diagnóstico, no qual foram utilizadas 256 amostras de soro: 79 (30,9%) obtidas de indivíduos microfilarêmicos e 177 (60,1%), de indivíduos amicrofilarêmicos e que testaram negativo para os testes imunológicos Bm14 CELISA e Og4C3 ELISA. A definição do ponto de corte ideal, bem como da acurácia do kit Filaria Detect™ IgG4, foi obtida através da construção de curvas ROC, sendo a densidade óptica de 0,239 aquela na qual o teste obteve melhor desempenho, com sensibilidade de 81,0% e especificidade de 96,6%. Os resultados obtidos demonstraram que o kit Filaria Detect™ IgG4 é uma ferramenta promissora para investigação e monitoramento de áreas submetidas ao tratamento em massa para filariose linfática.

## Abstract (en)

The Global Programme to Eliminate Lymphatic Filariasis, launched by the World Health Organization in the year 2000, proposes the use of circulating filarial antigen tests as a diagnostic tool to assess and monitor initiatives to control filarial infection. However, despite a high sensitivity, these tests are not efficient to detect infection at early stages, before worms have reached the adult stage. Considering this limitation, anti-filarial antibody testing has been suggested as an alternative, given that the antibodies produced against the larvae are detectable before the presence of circulating filarial antigen. The objective of the present study was to determine the diagnostic cut-off and the accuracy of the Filaria Detect™ IgG4 kit employing recombinant Wb123 antigen for diagnosis of lymphatic filariasis in Brazil. For that, we performed a diagnostic evaluation study in which 256 serum samples were analyzed: 79 (30.9%) obtained from microfilaremic individuals and 177 (60.1%) from amicrofilaremic individuals who tested negative with the Bm14 CELISA and Og4C3 ELISA immunologic tests. The ideal cutoff as well as the Filaria Detect™ IgG4 kit accuracy were determined based on ROC curve analyses, with an optical density of 0.239 identified as the cutoff with the best performance, with 81.0% sensitivity and 96.6% specificity. The results show that the Filaria Detect™ IgG4 kit is a promising tool for investigation and monitoring of areas undergoing mass drug administration for lymphatic filariasis.

## RESUMEN

En el programa mundial de eliminación de la filariasis linfática, puesto en marcha por la Organización Mundial de la Salud en el año 2000, se propone el uso de pruebas de detección del antígeno filárico circulante como instrumento de diagnóstico para la evaluación y el seguimiento de las medidas de control de la parasitosis. Sin embargo, esas pruebas, a pesar de tener un alto grado de sensibilidad, no permiten detectar con eficiencia la infección en su fase inicial, cuando todavía no existen helmintos adultos. En vista de esa limitación, se ha señalado como una opción el estudio de anticuerpos antifiláricos, puesto que los anticuerpos producidos contra las larvas infectantes del parásito se detectan antes de la existencia de antígeno filárico circulante. El objetivo de este estudio fue definir el punto de corte y evaluar la exactitud del estuche Detect™ para pruebas de anticuerpos antifiláricos IgG4, fabricado con el antígeno recombinante Wb123, para el diagnóstico de la filariasis linfática en Brasil. Para ello, se realizó un estudio de evaluación de la prueba diagnóstica, en el cual se utilizaron 256 muestras de suero, a saber, 79 (30,9%) obtenidas de personas microfilarémicas y 177 (60,1%) de personas amicrofilarémicas, que arrojaron resultados seronegativos en las pruebas inmunológicas CELISA Bm14 y ELISA Og4C3. La definición del punto de corte ideal y de la exactitud del estuche Detect™ se obtuvo con la construcción de curvas de la característica operativa del receptor (ROC); una densidad óptica de 0,239 marcó el mejor nivel de desempeño de la prueba, con una sensibilidad de 81,0% y una especificidad de 96,6%. Los resultados obtenidos demostraron que el estuche Detect™ es un instrumento prometedor para la investigación y el seguimiento de las regiones donde se realiza un tratamiento masivo de la filariasis linfática.

A filariose linfática, doença endêmica em 72 países das regiões tropicais e subtropicais, afeta cerca de 120 milhões de pessoas em todo o mundo (1). Essa doença tem como agentes etiológicos os nematoides das espécies *Brugia malayi, Brugia timori* e *Wuchereria bancrofti* (1) e é considerada pela Organização Mundial da Saúde (OMS) uma das principais causas mundiais de incapacidade permanente, tendo repercussão econômica e psicossocial na vida de seus portadores (1). No Brasil, a filariose linfática é causada exclusivamente pela espécie *W. bancrofti*, endêmica no estado de Pernambuco, particularmente na Região Metropolitana do Recife, que abrange os municípios de Recife, Olinda, Jaboatão dos Guararapes e Paulista (2).

Em 1997, a OMS elegeu a filariose linfática como uma das seis doenças infecciosas potencialmente erradicáveis, determinando, através da resolução 50.29, a adoção de metas para eliminação global da doença até o ano de 2020 (3). Em resposta a essa resolução, o Plano Global de Eliminação da Filariose Linfática (PGEFL) foi criado no ano 2000, tendo como pilares a administração massiva de medicamentos (AMM) antifilariais, o controle da morbidade filarial e o desenvolvimento de novas ferramentas de diagnóstico que pudessem ser utilizadas na avaliação e na vigilância de áreas endêmicas submetidas às ações do PGEFL (1, 4). Nesse mesmo ano, o Brasil criou o Programa Nacional de Eliminação da Filariose Linfática (PNEFL), cujas atividades são apoiadas pela Resolução 190/96 do Conselho Nacional de Saúde (45).

Dados recentes apontam que a AMM atingiu uma cobertura global acima de 7 bilhões de tratamentos nos países onde a filariose linfática é endêmica, e que aproximadamente 24 países já completaram cinco ciclos de tratamento e estão sob vigilância pós-AMM para demonstrar que a eliminação foi alcançada (6). De acordo com as recomendações da OMS, a vigilância das áreas pós-AMM deve ser realizada através do teste de detecção de antígenos circulantes filariais (ACF). Embora o teste tenha alta sensibilidade para o reconhecimento dos antígenos dos vermes adultos, não reconhece a infecção na sua fase inicial, visto que os antígenos são produzidos mais tardiamente do que os anticorpos específicos (1, 4, 6). Assim, o diagnóstico baseado na pesquisa de anticorpos pode ser mais adequado na identificação precoce da infecção filarial, além de poder ser usado como marcador de endemicidade residual ou do início de um ressurgimento da transmissão, funcionando como um sistema de alerta em áreas que foram submetidas a medidas de erradicação da filariose linfática (7).

Com base na pesquisa de anticorpos filariais, vários testes foram desenvolvidos utilizando diversos antígenos recombinantes, com destaque para WbSXP-1 (8), Wb123 (9), BmR1, BmSXP, Bm33 (10, 11) e Bm14 (12). O Bm14 deu origem ao kit de captura de anticorpos mais amplamente utilizado para o diagnóstico de filariose linfática, o ELISA Bm14 (CELISA) (12-14). No entanto, problemas de reatividade cruzada foram relatados com o teste CELISA (12, 13); além disso, o fato de o antígeno recombinante ser derivado da *B. malawi* em vez da *W. bancrofti* (espécie responsável pela filariose linfática no Brasil) pode levar a resultados falsos negativos. O antígeno recombinante Wb123, produzido a partir de larvas infectantes (L_3_) de *W. bancrofti*, foi utilizado pela InBios International, Inc. no desenvolvimento de um kit diagnóstico denominado Filaria Detect™ IgG4 ELISA. Segundo estudos, ele funcionaria como um marcador precoce de exposição filarial (12, 13, 15). Em relação ao kit, o fabricante recomenda que, para melhorar o desempenho do ensaio, os países endêmicos estabeleçam seus próprios valores de corte para diagnóstico, utilizando amostras sorológicas locais coletadas de indivíduos testados positiva e negativamente para filariose linfática, a fim de garantir maior precisão nos resultados obtidos (16).

Com base nessas observações, o presente estudo teve como objetivo estabelecer o melhor valor de corte para o diagnóstico filarial com o kit Filaria Detect™ IgG4 (Wb123 ELISA) no Brasil, bem como definir a acurácia do teste.

## MATERIAIS E MÉTODOS

Foi realizado um estudo de avaliação de teste diagnóstico de fase II (17). Nesse tipo de estudo, utilizam-se amostras sabidamente positivas e negativas para a definição dos valores de sensibilidade e especificidade e para os valores preditivos positivos e negativos (VPP e VPN) do teste diagnóstico em análise. As amostras de soro utilizadas neste estudo foram obtidas do banco de amostras do Serviço de Referência Nacional em Filarioses do Instituto Aggeu Magalhães (IAM)/Fundação Oswaldo Cruz (Fiocruz), em Recife, no estado de Pernambuco. O estudo foi aprovado pelo Comitê de Ética em Pesquisa do IAM/Fiocruz (CAEE: 47227015.9.0000.5190).

Foram analisadas 256 amostras, sendo categorizadas em microfilarêmicas e amicrofilarêmicas de acordo com o estado da microfilaremia do paciente, obtido através da técnica parasitológica de filtração de sangue venoso noturno em membrana de policarbonato (FSV-MP), considerada o padrão ouro (18). Do total de amostras estudadas, 79 (30,9%) foram obtidas de pacientes diagnosticados como microfilarêmicos e 177 (60,1%), de pacientes amicrofilarêmicos e negativos para os testes imunológicos Bm14 CELISA e Og4C3 ELISA (12, 18). Do segundo grupo, 74 (41,8%) amostras foram obtidas de indivíduos residentes na Região Metropolitana de Recife, com a característica de também terem sido diagnosticados pela técnica de ultrassonografia como negativos para presença de helmintos adultos nos vasos linfáticos da bolsa escrotal e dos membros inferiores; por sua vez, 103 amostras (58,2%) foram obtidas de indivíduos residentes em Limoeiro (PE), área considerada não endêmica para filariose linfática.

Os testes imunológicos Og4C3 ELISA, Bm14 CELISA e Wb123 ELISA foram realizados conforme os protocolos dos kits, seguindo as instruções do fabricante (12, 18). A leitura da densidade óptica (DO) das placas dos testes ELISA foi realizada com comprimento de onda de 450 nm, com leitor de microplaca Thermo Scientific Multiskan FC.

Na análise dos dados, utilizaram-se duas técnicas estatísticas para determinar e avaliar o melhor ponto de corte para o Wb123 ELISA. Na primeira, uma curva ROC foi construída a partir dos valores de DOs do teste Wb123 ELISA, tendo como referência os resultados positivos e negativos provenientes da FSV-MP, sendo esse o controle. Na segunda técnica, também foi construída uma curva ROC; porém, foi utilizada uma análise de classe latente, que faz parte da família de modelos de equação estrutural. A partir dessa análise, foi criada uma variável latente (VL) baseada nos resultados (positivos/negativos) dos testes Og4C3 ELISA, Bm14 CELISA e Wb123 ELISA (19).

Com base no valor de corte para diagnóstico filarial obtido através da curva ROC (FSV-MP) e da VL, determinou-se a acurácia do teste a partir do cálculo de sensibilidade, especificidade, VPP, VPN, razão de probabilidade (ou razão de verossimilhança, positiva [RV+] ou negativa [RV-]) e área sob a curva (AUC) (19, 20). A análise da curva ROC foi realizada com o *software* MedCalc (v. 9.2). As análises estatísticas foram realizadas utilizando o programa R (v.3.3.2).

## RESULTADOS E DISCUSSÃO

**FIGURA 1.**
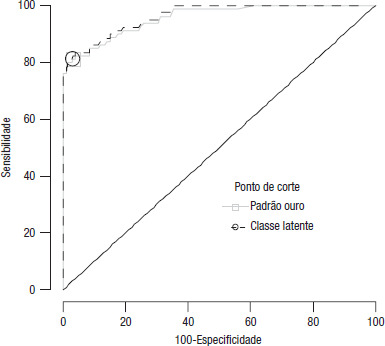
Sobreposição das curvas ROC obtidas com filtração de sangue venoso por membrana de policarbonato (padrão ouro) e variável latente, ponto de corte 0,239

A construção da curva ROC para o Wb123 ELISA, tendo como referência a FSV-MP (padrão ouro), apontou como ponto de corte do teste uma DO de 0,215. No entanto, ao analisarmos os resultados de outros parâmetros (AUC, VPP, VPN, RV+, RV-), chegamos à definição de um valor de DO de 0,239 como ponto de corte otimizado da positividade para infecção filarial com o Wb123 ELISA, com sensibilidade de 81,0%, especificidade de 96,6%, RV+ de 23,9 e RV- de 20,0. Esses resultados foram semelhantes aos observados para a curva ROC criada utilizando a VL quando aplicado o ponto de corte correspondente à DO de 0,240 (tabela 1). Na análise comparativa das duas curvas ROC construídas, houve sobreposição entre as curvas, com AUC de 0,96 (intervalo de confiança de 95%: 0,923; 0,978) (figura 1).

**TABELA 1.** Acurácia do teste Wb123 ELISA para detecção de filariose obtida através da curva ROC utilizando como referência a filtração de sangue venoso por membrana de policarbonato (FSV-MP) e a variável latente (VL)

Ponto de corte (densidade óptica)	Sensibilidade (IC95%)	Especificidade (IC95%)	Razão de verossimilhança positiva (IC95%)	Razão de verossimilhança negativa (IC95%)	Valor preditivo positivo (IC95%)	Valor preditivo negativo (IC95%)
FSV-MP > 0,239	81,01 (70,6; 89,0)	96,61 (92,8; 98,7)	23,90 (17,1; 33,4)	20,00 (17,2; 22, 4)	91,43 (82,5; 96,0)	91,94 (87,1; 95,0)
VL > 0,240	81,00 (71,0; 88,1)	96,60 (92,8; 98,4)	23,82 (17,1; 33,3)	19,65 (17,2; 22,2)	91,43 (82,5; 96,1)	91,94 (87,1; 95,0)

De acordo com Greiner et al. (21) e Hajian-Tilaki (22), a curva ROC, quando combinada com outros valores de precisão de um teste (sensibilidade, especificidade, AUC, RV+, RV-), permite otimizar a decisão acerca do ponto de corte ideal. Portanto, embora o ponto de corte para Wb123 ELISA tenha sido identificado na curva ROC (FSV-MP) como 0,215 DO (grupo controle), a análise conjunta de outros parâmetros de precisão indicou que o melhor ponto de corte na nossa região é de 0,239 DO.

A técnica da curva ROC tem sido amplamente utilizada na epidemiologia clínica para avaliar a capacidade diagnóstica dos marcadores. As medidas de precisão derivadas da curva ROC, como a AUC, não são distorcidas pelas flutuações causadas por critérios de decisão arbitrários ou cortes. A AUC determina a capacidade inerente de um teste para discriminar entre populações doentes e saudáveis. Quando a AUC é igual a 1, o teste diagnóstico distingue perfeitamente o paciente do não paciente. Identificou-se uma AUC significativamente elevada (AUC 0,96; *P* < 0,01) para a curva ROC Wb123 ELISA, demonstrando o potencial do teste para discriminar indivíduos positivos e negativos (19, 20).

A metodologia aplicada no cálculo da curva ROC buscou garantir que as medidas de desempenho obtidas fossem precisas, não tendenciosas e corretamente estimadas e apresentadas. Os valores de sensibilidade e especificidade, combinados com a AUC, demonstram a validade inerente ao teste diagnóstico (21, 22). Os VPP e VPN encontrados para o Wb123 ELISA foram elevados: 91,4% e 91,9%, respectivamente. O VPP representa a proporção de resultados positivos entre os indivíduos verdadeiros positivos, enquanto o VPN representa a proporção de desfechos negativos que são verdadeiros negativos (21, 22).

Houve certa dificuldade em encontrar na literatura trabalhos semelhantes ao presente estudo ou passíveis de comparação, uma vez que nenhum trabalho anterior foi realizado no Brasil ou com amostras brasileiras. Encontraram-se estudos de regiões endêmicas diferentes, nas quais a prevalência para filariose linfática e as características da população e dos parasitas diferem do Brasil (15, 16).

Em conclusão, os resultados obtidos apontam a DO de 0,239 como ponto de corte ideal a ser empregado em amostras sorológicas obtidas de pacientes residentes em território brasileiro quando empregado o kit diagnóstico Filaria Detect™ IgG4 (Wb123 ELISA). Com esse ponto de corte, o kit apresentou o melhor desempenho, com percentuais elevados de sensibilidade e especificidade, o que demonstra o potencial do kit como ferramenta promissora para investigação e monitoramento de áreas submetidas a AMM para filariose linfática, contribuindo para o Plano Global de Eliminação da doença.

## Declaração.

As opiniões expressas no manuscrito são de responsabilidade exclusiva dos autores e não refletem necessariamente a opinião ou política da RPSP/PAJPH ou da Organização Pan-Americana da Saúde (OPAS).****
